# A dual 6‐month (stage 1) & 18‐month (stage 2) prospective, randomized, placebo‐controlled, double‐blind, pivotal clinical trial investigating efficacy and safety of buntanetap in early Alzheimer's patients

**DOI:** 10.1002/alz70861_108764

**Published:** 2025-12-23

**Authors:** Sarah J MacCallum, Melissa Gaines, Cheng Fang, Matthew J Peterson, Alexander Morin, Maria L. Maccecchini

**Affiliations:** ^1^ Annovis Bio, Malvern, PA USA

## Abstract

**Background:**

Annovis Bio initiated a new pivotal clinical trial in early Alzheimer's disease (AD). This Phase III trial is being conducted to determine whether buntanetap, which inhibits the translation of neurotoxic aggregating proteins, is able to a) duplicate the efficacy seen in our phase IIb study and improve symptomatic effects and b) show potential disease‐modifying effects.

**Method:**

The study started in March 2025 and is progressing at a good pace. In total, 760 participants will be randomized across up to 100 sites. The study is being conducted at both academic and private sites across the US. Participants are being recruited through sites' databases, physician referrals, and word of mouth. We will collect and summarize data as to site opening, patient inclusion using the plasma pTau217 biomarker, and participant enrollment.

**Result:**

Tables will be featured on the poster showing the status of the following per July 2025:

• An update of the recruiting sites and locations

• A US map with all site locations shown as pins, to highlight regional study access

• pTau217 selection inclusion/exclusion figures

• Current enrollment versus Target enrollment of 760 participants

• Actual monthly participant accrual versus projected monthly accrual

• Estimated timelines for the 6‐month and 18‐month end of study data readouts

• Screen failure rate with breakdown of screen failure reasons

**Conclusion:**

In summary, we will show the ease of using plasma biomarkers to determine the presence of AD. We will provide updates on total enrollment and the 6‐ and 18‐month data readout timepoints.

